# Monitoring the Concentrations of Na, Mg, Ca, Cu, Fe, and K in *Sargassum fusiforme* at Different Growth Stages by NIR Spectroscopy Coupled with Chemometrics

**DOI:** 10.3390/foods14010122

**Published:** 2025-01-03

**Authors:** Sisi Wei, Jing Huang, Ying Niu, Haibin Tong, Laijin Su, Xu Zhang, Mingjiang Wu, Yue Yang

**Affiliations:** Zhejiang Provincial Key Laboratory for Water Environment and Marine Biological Resources Protection, College of Life and Environmental Science, Wenzhou University, Wenzhou 325035, China; weisisi1511@163.com (S.W.); jinghuang0224@163.com (J.H.); naive916@163.com (Y.N.); hbtong@wzu.edu.cn (H.T.); sulj@wzu.edu.cn (L.S.); zhangx@wzu.edu.cn (X.Z.); wmj@wzu.edu.cn (M.W.)

**Keywords:** near-infrared spectroscopy, chemometrics, minerals, growth stage, *Sargassum fusiforme*

## Abstract

*Sargassum fusiforme*, an edible seaweed, plays a crucial role in our daily lives by providing essential nutrients, including minerals, to the human body. The detection of mineral content during different growth stages of *S. fusiforme* benefits the goals of ensuring product quality, meeting diverse consumer needs, and achieving quality classification. Currently, the determination of minerals in *S. fusiforme* primarily relies on inductively coupled plasma mass spectrometry and other methods, which are time-consuming and labor-intensive. Thus, a rapid and convenient method was developed for the determination of six minerals (i.e., Na, Mg, Ca, Cu, Fe, and K) in *S. fusiforme* via near-infrared (NIR) spectroscopy based on chemometrics. This study investigated the variations in minerals in *S. fusiforme* from different growth stages. The effects of four spectral pretreatment methods and three wavelength selection methods, including the synergy interval partial least squares (SI-PLS) algorithm, genetic algorithm (GA), and competitive adaptive reweighted sampling method (CARS) on the model optimization, were evaluated. Superior CARS-PLS models were established for Na, Mg, Ca, Cu, Fe, and K with root mean square error of prediction (*RMSEP*) values of 0.8196 × 10^3^ mg kg^−1^, 0.4370 × 10^3^ mg kg^−1^, 1.544 × 10^3^ mg kg^−1^, 0.9745 mg kg^−1^, 49.88 mg kg^−1^, and 7.762 × 10^3^ mg kg^−1^, respectively, and coefficient of determination of prediction (*R_P_*^2^) values of 0.9787, 0.9371, 0.9913, 0.9909, 0.9874, and 0.9265, respectively. *S. fusiforme* demonstrated higher levels of Mg and Ca at the seedling stage and lower levels of Cu and Fe at the maturation stage. Additionally, *S. fusiforme* exhibited higher Na and lower K at the growth stage. NIR combined with CARS-PLS is a potential alternative for monitoring the concentrations of minerals in *S. fusiforme* at different growth stages, aiding in the convenient evaluation and further grading of the quality of *S. fusiforme*.

## 1. Introduction

*Sargassum fusiforme* (Harv.) Setchel, which belongs to the Sargassaceae family, is an economically important seaweed food and tonic herb [1]. Rich in minerals, polysaccharides, polyphenols, proteins, dietary fiber, and other active ingredients, *S. fusiforme* is an excellent choice for the human body to supplement essential elements [2,3,4,5,6]. It exerts various pharmacological effects, including antioxidant, anti-Alzheimer’s disease, antitumor activities, lowering blood glucose, and blood lipid levels, etc. [7,8,9,10]. In addition, it also boosts human immunity and maintains hair luster and skin lubrication [11]. With the growing trend of “return to nature,” increasing attention has been paid to *S. fusiforme*, leading to the gradual expansion of the demand and the expansion of the planting scale year by year. In 2022, the cultivation area and the total output value of *S. fusiforme* in China reached 1595 hectares and 33,372 tons, respectively, reflecting a growth rate of 10.92% and 7.2% from the previous year [12].

Based on their relation to body weight, minerals can be divided into macro minerals (with contents exceeding 0.01% of body weight) and trace minerals (with contents less than 0.01% of body weight) [13]. Macro minerals, such as sodium (Na), magnesium (Mg), calcium (Ca), and potassium (K), along with trace minerals, like copper (Cu) and iron (Fe), are also crucial for the physiological activity, composition, and structure of the human body. For instance, Ca and Mg, the essential constituents of bones and teeth, also play crucial roles in various enzymatic reactions, protein synthesis reactions, blood sugar regulation, and other physiological activities [13]. Fe, as the source of hemoglobin, is involved in hematopoiesis and oxygen transport [14]. Therefore, the mineral content of *S. fusiforme* has gained increased attention from consumers as a key quality indicator.

The mineral content can be significantly influenced by various factors throughout the entire growth stage, including but not limited to climate conditions, geographical origin, temperature variations, and water quality [15]. Consequently, the accumulation of minerals is inevitably influenced by different growth stages, thereby resulting in variations in the quality of *S. fusiforme*. Now, the traditional methods for the determination of mineral content in *S. fusiforme* include ultraviolet spectrophotometry, atomic fluorescence spectrometry, atomic absorption spectrophotometry, and inductively coupled plasma mass spectrometry (ICP-MS). Although these methods are stable and accurate, they are cumbersome, costly, and environmentally hazardous. Therefore, there is an urgent need for the development of a rapid and convenient method to detect the mineral content of *S. fusiforme.*

Near-infrared (NIR) spectroscopy offers a promising alternative for rapid substance determination. The principle behind NIR spectroscopy lies in the vibration of O-H, N-H, C-H, and other hydrogen-containing groups within organic matter when exposed to NIR light. This generates characteristic information, including the overtones and combined frequency of vibration. This information combines to reflect the physical properties, chemical composition, and other details of a material [16]. Although minerals do not exhibit characteristic absorption peaks in the NIR spectrum, their information can be indirectly represented through their association with organic matter [17]. Several studies have been conducted on the analysis of mineral contents in crops and algae, yielding promising findings. Neto et al. accurately predicted the content of selected minerals, including the K, Na, Mg, and Na/K ratio in sugarcane juice using visible/NIR spectra [18]. The coefficients of determination (*R*^2^) of external validation were 0.78, 0.89, 0.93, and 0.74, respectively. Cozzolino and Moron successfully determined the contents of Na, S, B, Zn, Mn, Cu, and Fe in white clover and lucerne from different locations [19]. The coefficient of determination of calibration (*R*^2^*_C_*) of the modified partial least squares regression models exceeded 0.75, except for that of Fe. The model’s accuracy may not reach exceptional levels, but it is adequate for its utilization as an auxiliary tool in agricultural production. Liu et al. proposed a novel method called the constrained difference extreme learning machine, which utilizes NIR to identify Zn, Pb, Cd, and Cu in mussels [20]. The results demonstrated a detection accuracy of over 95% for the four substances, thereby establishing the proposed method as highly desirable for the quality assessment of mussels. However, to the best of our knowledge, no study has been conducted on the determination of mineral contents in *S. fusiforme* using NIR.

Our primary motivation was to utilize NIR to monitor the concentrations of six selected minerals (Na, Mg, Ca, Cu, Fe, and K) in *S. fusiforme* at different growth stages in order to provide a quick and effective method for the agricultural production and quality control of *S. fusiforme*. The specific objectives of this study were to (1) investigate the potential of NIR spectroscopy in the quantitative determination of contents of Na, Mg, Ca, Cu, Fe, and K in *S. fusiforme*; (2) investigate the concentration differences in minerals in *S. fusiforme* at different growth stages; and (3) compare the optimization effects of three wavelength selection methods, namely the synergy interval partial least square (SI-PLS), genetic algorithm (GA), and competitive adaptive reweighted sampling method (CARS), for mineral quantitative models of *S. fusiforme*.

## 2. Materials and Methods

### 2.1. Sampling and Sample Preparation

The experimental samples included seven batches of *S. fusiforme* artificially cultivated in Dongtou District, Wenzhou City, Zhejiang Province, China. To investigate the variations in mineral contents during development, seven batches were harvested, including the seeding stage (11 December 2020 and 9 January 2021), growth stage (1 February 2021, 5 March 2021, and 20 March 2021), and maturity stage (4 April 2021 and 19 April 2021). Each batch contained 20 *S. fusiforme* samples with similar appearance and size, and a total of 140 samples were collected for investigation.

The *S. fusiforme* samples were transported to the laboratory for immediate processing. Various impurities, such as silt, gravel, and attached organisms, were removed by rinsing all samples with water. Then, the samples were dried to constant weight at 80 °C and ground into a fine powder, which was screened through an 80-mesh sieve. The powdered samples were stored at room temperature for later use.

### 2.2. NIR Spectral Acquisition

The NIR spectra of the samples were obtained by spreading 2 g of sample powder in a rotating cup and scanning it 64 times in the range of 4000 cm^−1^ to 10,000 cm^−1^ with air as the background. The resolution was set at 8 cm^−1^. Three spectra were collected for each sample, and the average spectrum was used for subsequent analysis. The scanning instrument used was a Fourier transform-NIR spectrometer equipped with an integrating sphere (Antaris II, Thermo Fisher Scientific, Waltham, MA, USA). Mineral content was determined using an inductively coupled plasma mass spectrometer (iCAP TQ ICP-MS, Thermo Fisher Scientific, Waltham, MA, USA). The instrument operated at a plasma power of 1550 W. The operating conditions were meticulously controlled, with the atomizing gas flow rate set to 0.80 L min^−1^, the auxiliary gas flow rate also at 0.80 L min^−1^, and the cooling gas flow rate at 14.0 L min^−1^. The pump speed was maintained at a steady 40 r min^−1^. The isotopes used were ^23^Na, ^24^Mg, ^44^Ca, ^63^Cu, ^57^Fe, and ^39^K.

### 2.3. Reference Assays

The content of minerals, such as Na, Mg, Ca, Cu, Fe, and K, in *S. fusiforme* was determined using wet digestion with ICP-MS [21,22]. A mixed standard solution of Na, Mg, Ca, Cu, Fe, and K was prepared in 5% HNO_3_ and stored in a refrigerator at 4 °C. Then, 0.5 g of sample powder was placed in an ablation cup with 1 mL pure water and 8 mL concentrated HNO_3_, and the mixture stood at ambient temperature for 12 h to allow for the complete reaction of the sample powder. Then, the ablation cups were transferred to an electric heating plate for digestion. After that, the test material was obtained by transferring the cooled digestion solution to a volumetric flask and then diluting it with 5% HNO_3_.

The dissolution process was carried out as follows: The temperature was raised from ambient temperature to 130 °C and held for 1.5 h. During this period, the ablation cup was replenished with 1 mL of HNO_3_ every 20 min to prevent the sample from being heated into carbon. Then, 1 mL of HClO_4_ was added to the cups, and the temperature was raised to 180 °C to remove the remaining carbon. The heating was stopped when about 1 mL of solution remained in the cups.

The content of the above minerals in each sample was determined three times via ICP-MS, and the average value obtained was considered the reference value.

### 2.4. Wavelength Selection Algorithms

SI-PLS was proposed based on the interval partial least squares method. In this algorithm, the entire spectrum range is divided into several subintervals of equal sizes, and then a PLS model is established by combining all possible combinations of 2, 3, and 4 subintervals [23]. The optimal combination was determined based on the lowest *RMSECV*.

The GA is a random-search global optimization method based on Darwin’s theory of biological evolution, which utilizes random search to simulate the natural selection and genetic evolution of organisms [24]. All the possible solutions of a problem are compared to an infinite number of chromosomes, and iterations are performed until a condition is reached where the iteration has stopped. Each iteration involves evaluating the chromosomes in terms of “fitness” and selecting those with high “fitness” for duplication, crossover, and mutation to generate new chromosomes for the next iteration. The *RMSECV* was calculated to evaluate the importance of the wavelength set, and the one with the lowest *RMSECV* was considered optimal.

The CARS method consists of four successive steps in each iteration, including Monte Carlo sampling, exponentially decreasing function (EDF), adaptive reweighted sampling function (ARS), and the *RMSECV* calculation. The PLS model is established by a proportion of samples randomly selected by Monte Carlo sampling. The EDF and ARS were subsequently utilized to eliminate wavelengths with lower absolute values of regression coefficients, as these wavelengths provide limited or negligible information [25]. The *RMSECV* of each PLS model was then calculated, and the wavelengths included in the model with the smallest *RMSECV* were finally selected as the key ones.

### 2.5. Model Performance Evaluation

In this study, coefficients of determination for calibration (*R_C_*^2^) and prediction (*R_P_*^2^), as well as the root mean square error for calibration (*RMSEC*) and prediction (*RMSEP*) were chosen as the evaluation criteria for model performance. Generally, a more robust and accurate model is achieved when *R_C_*^2^ and *R_P_*^2^ are closer to 1, while the *RMSEC* and *RMSEP* are smaller and closer.

## 3. Results and Discussion

### 3.1. NIR Spectral Features

**Figure 1:**
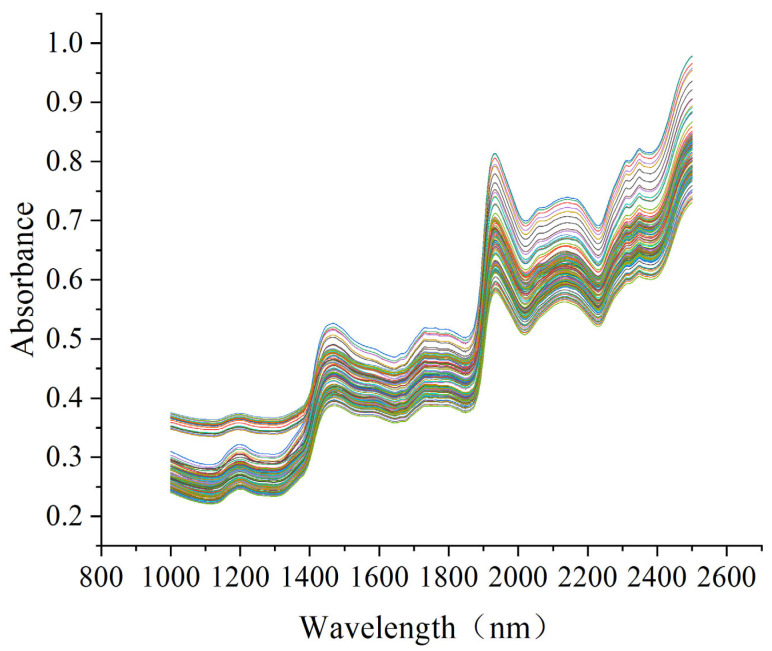
Raw near-infrared spectra of *Sargassum fusiforme* samples. Each line represents the near-infrared spectrum of each sample.

Figure 1 shows the raw NIR spectra of 140 samples ranging from 1000 to 2500 nm. Upon excitation by NIR light, the hydrogen-containing groups in the organic matter go through stretching or bending vibrations, which generate overtones and combined frequency information and form the NIR spectra [26]. The NIR spectra of the samples exhibited an evident absorption peak at around 1440 nm, which was primarily attributed to the first overtone of O-H [27]. Another strong absorption peak was observed at 1930 nm, which was associated with the combination of the stretching and deformation of O-H bonds [28]. In addition, broad absorption bands were located at 1720 and 2250 nm, which are related to the vibrations of C-H and CH_2_ groups, as well as the stretching and deformation combination of C-H [29].

### 3.2. Outlier Detection and Sample Partition

**Figure 2:**
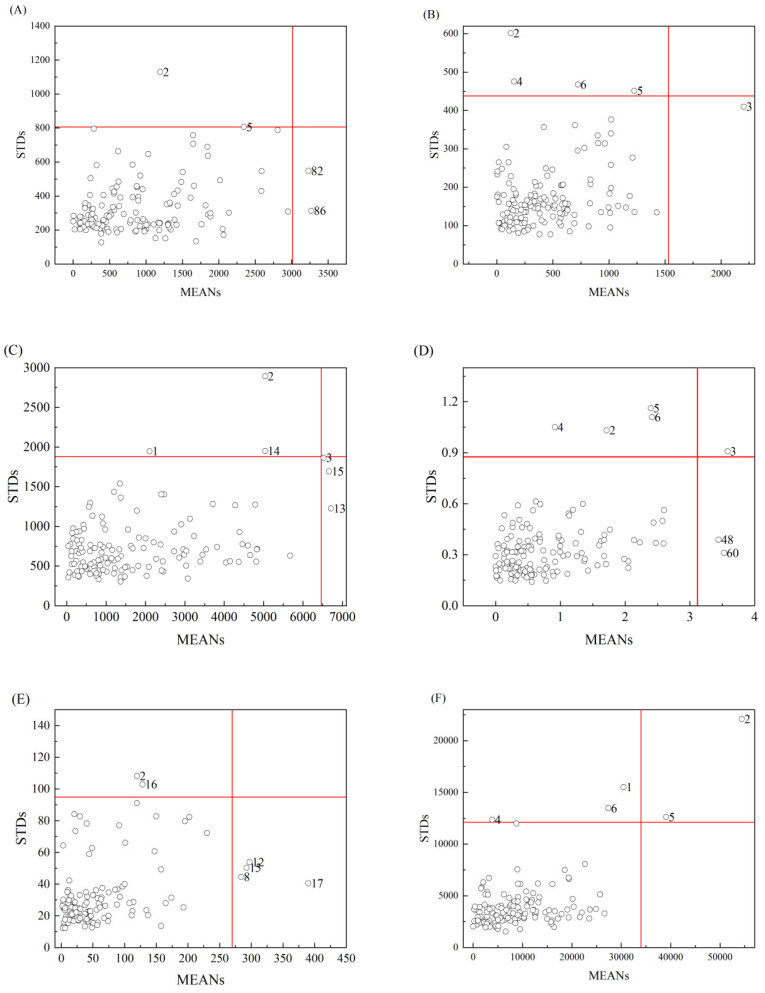
Scatter plots of mean values (MEANs) and standard deviations (STDs) of prediction errors for Na (**A**), Mg (**B**), Ca (**C**), Cu (**D**), Fe (**E**), and K (**F**).

During the collection of sample spectra or reference analyses, abnormal samples inevitably arise due to objective factors, such as environmental variations, or subjective ones, like human operation. It is necessary to eliminate these abnormal samples before modeling to ensure the prediction accuracy [30,31]. In this study, Monte Carlo cross-validation (MCCV) was used to identify abnormal samples. A total of 2500 iterations of sampling were conducted, and 80% of the samples were randomly selected as the calibration set to construct a PLS model at each iteration. The standard deviations (STDs) and mean values (MEANs) were obtained, and the corresponding scatter plots of Na, Mg, Ca, Cu, Fe, and K were shown in Figure 2. The M_threshold_ represents the threshold value of MEANs, and S_threshold_ represents the threshold value of STDs. Samples exceeding either the M_threshold_ or the S_threshold_ will be considered as abnormal samples and removed. According to the threshold formula [32], the M_threshold_ and S_threshold_ were 3012 and 806.3 for Na, 1532 and 438.0 for Mg, 6461 and 1878 for Ca, 3.116 and 0.8755 for Cu, 269.6 and 94.95 for Fe, and 33,970 and 12,130 for K, respectively. As shown in Figure 2, each scatter represents a sample, the red line represents the threshold, and the number represents the serial numbers of the abnormal sample. It can be seen that the six mineral samples were relatively clustered, and most of them were within the red line, especially Mg, Ca, Cu, and K. A few samples scattered outside the red line were considered abnormal, which could decrease the performances of the calibration models. As shown in Figure 2, there were 4, 5, 6, 7, and 5 samples found outside the red line for Na, Mg, Ca, Cu, and K, respectively. These samples were identified as abnormal and removed. Fe cannot be detected in the sixth batch of samples, and there were 6 abnormal samples. Finally, there were 136, 135, 134, 133, 114, and 135 samples remaining for the subsequent modeling of Na, Mg, Ca, Cu, Fe, and K, respectively.

After removing the abnormal samples, the remaining ones were divided into calibration and prediction sets using the Kennard and Stone (KS) algorithm. The KS algorithm is applied to calculate the Euclidean distance between each pair of samples through multiple iterations and continuously select the sample pair with the largest Euclidean distance into the calibration set until reaching the desired number of samples [33]. Here, the ratio of calibration and prediction samples was set to 4:1, and the statistical description of the reference values of sample sets was presented in Table 1. The reference values in the calibration set covered those in the prediction set, which is conducive to the establishment of models with high accuracy and stability [34].

### 3.3. Comparison of Different Spectral Preprocessing Methods

Although these samples were crushed and screened before spectrum acquisition to minimize its influence on the model, certain factors, such as a low signal-to-noise ratio, baseline drift, and significant spectral overlap, still existed during the collection of sample spectral and chemical information, potentially influencing the robustness of the model [35,36]. Therefore, spectral preprocessing must be conducted before modeling. The first derivative Savitzky–Folay (1D/SG) effectively eliminates baseline drift and enhances resolution, while the smooth algorithm reduces noise and improves the signal-to-noise ratio of the spectrum [37]. Both the standard normalized variate (SNV) and multiplicative scatter correction (MSC) can minimize the effect of scattered light on the spectrum and remove background signals [37]. Here, smooth, 1D/SG, MSC, and SNV methods were used to pretreat the original spectra (Table 2). According to the values of *R_C_*^2^, *R_P_*^2^, the *RMSEC,* and the *RMSEP* in Table 2, MSC and 1D/SG were the optimal pretreatment methods for Cu and Na, respectively. For Fe and K, the optimal preprocessing method was smooth. Regarding Mg and Ca, the model built on the raw spectra exhibited better performances than those on the other methods.

### 3.4. PLS Models Based on Different Wavelength Selection Algorithms

#### 3.4.1. Performance of the Full-PLS Models

The Full-PLS model was often used as a benchmark to investigate the efficiency of wavelength selection methods. Here, the full spectrum within the range of 1000 nm to 2500 nm contained 1557 wavelengths. In PLS, the principal component analysis was employed to select the latent variables (LVs), which plays a critical role in ensuring model accuracy and stability. The optimal number of LVs was determined via tenfold cross-validation, and Full-PLS models were established for Na, Mg, Ca, Cu, Fe, and K (Table 3). Generally, all the six Full-PLS models showed good performances, with all *R*^2^ reaching 0.9 and a relatively small *RMSEP* being observed. Specifically, the Full-PLS model for Na, Ca, Cu, and Fe exhibited better performances, with both *R_C_*^2^ and *R_P_*^2^ being greater than 0.97. The Full-PLS models for Mg and K were relatively poorer (*R_C_*^2^ = 0.9729, *RMSEC* = 0.4427 × 10^3^ mg kg^−1^, *R_P_*^2^ = 0.9182, *RMSEP* = 0.4985 × 10^3^ mg kg^−1^ for Mg; *R_C_*^2^ = 0.9285, *RMSEC* = 6.905 × 10^3^ mg kg^−1^, *R_P_*^2^ = 0.9153, *RMSEP* = 8.330 × 10^3^ mg kg^−1^ for K).

#### 3.4.2. Performance of SI-PLS Models

In addition to the wavelengths associated with target information in the NIR spectra, a large number of irrelevant wavelengths were detected. The irrelevant information contained in the full spectrum can decrease the model performance [38], and the wavelength selection methods were thus used to identify the useful wavelengths that can capture the characterization information and structural characteristics of the target substance. Here, three wavelength selection methods, namely GA, SI-PLS, and CARS, were employed; the PLS models based on these methods were compared, and the results were also listed in Table 3.

In SI-PLS, the full spectrum was divided into 11–20 subintervals of equal size in sequential order. Then, 2, 3, and 4 subintervals were randomly combined to establish PLS models. Take Na as an example; the performances of PLS models based on different subinterval combinations were compared, and the results are presented in Table 4. It was observed that the optimal SI-PLS model was achieved by dividing the full spectrum into 19 subintervals and using the interval combination of “2, 3, 4, 6”. These selected wavelengths were distributed in the ranges of 1697–1792 nm and 1900–2317 nm. It was clear that these six minerals all obtained better performance corresponding to Full-PLS models. Specifically, the prediction performance for Na, Mg, Cu, and Fe has been improved considerably as *R_P_*^2^ increased from 0.9715, 0.9182, 0.9884, and 0.9826 to 0.9764, 0.9348, 0.9903, and 0.9873, respectively. Meanwhile, the corresponding *RMSEP* decreased from 0.9481 × 10^3^ mg kg^−1^, 0.4985 × 10^3^ mg kg^−1^, 1.0980 mg kg^−1^, and 58.70 mg kg^−1^ to 0.8632 × 10^3^ mg kg^−1^, 0.4451 × 10^3^ mg kg^−1^, 1.0034 mg kg^−1^, and 50.08 mg kg^−1^, respectively. As for Ca and K, there has been a certain improvement, with *R_P_*^2^ increasing to 0.9902 and 0.9190 and *RMSEP* decreasing to 1.636 × 10^3^ mg kg^−1^ and 8.149 × 10^3^ mg kg^−1^, respectively. In addition, the numbers of wavelengths screened by SI-PLS were reduced to 328 (Na), 367 (Mg), 347 (Ca), 390 (Cu), 415 (Fe), and 328 (K), which significantly simplified the model.

#### 3.4.3. Performance of the GA-PLS Models

**Figure 3:**
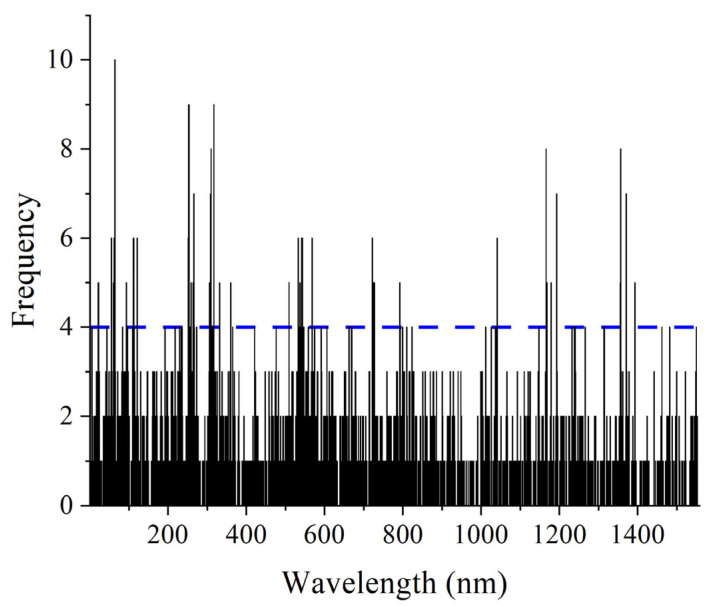
The histogram of selection frequencies for each wavelength after 100 runs by the genetic algorithm for Na. The blue dashed line indicates the boundary.

In the procedure of the GA, the number of iterations was set to 100, and 80% of samples were randomly selected to construct the PLS model in each iteration. The selection frequency histogram for Na was plotted in Figure 3, with the wavelengths represented on the X-axis and the selection frequency on the Y-axis. In each iteration, the frequency is increased by 1 when the corresponding wavelength is selected. The wavelengths with high cumulative frequencies are considered to be the key ones. It can be seen from Figure 3 that the wavelengths with frequencies greater than or equal to 4 were ultimately chosen. Finally, the GA selected 92, 85, 103, 98, 85, and 72 wavelengths for Na, Mg, Ca, Cu, Fe, and K, respectively. Take Na as an example; the selected wavelengths were mainly distributed in the ranges of 1003–1187 nm, 1395–1475 nm, 1610–1780 nm, 1895–2111 nm, and 2241–2490 nm. Based on the optimal wavelengths, the PLS models were constructed, and the results are also listed in Table 3. Compared to Full-PLS models, all six GA-PLS models exhibited improved performance to some extent. For Na, Cu, and Fe, the GA-PLS models yielded better performance, with the *R_P_*^2^ increasing from 0.9715, 0.9884, and 0.9826 to 0.9768, 0.9897, and 0.9849, respectively, while the *RMSEP* decreased from 0.9481 × 10^3^ mg kg^−1^, 1.0980 mg kg^−1^, and 58.70 mg kg^−1^ to 0.8557 × 10^3^ mg kg^−1^, 1.0360 mg kg^−1^, and 54.59 mg kg^−1^, respectively. The improvements for Mg, Ca, and K were small but still greater than those of the Full-PLS model (*R_P_*^2^ = 0.9216, *RMSEP* = 0.4881 × 10^3^ mg kg^−1^ for Mg; *R_P_*^2^ = 0.9900, *RMSEP* = 1.660 × 10^3^ mg kg^−1^ for Ca; *R_P_*^2^ = 0.9169, *RMSEP* = 8.251 × 10^3^ mg kg^−1^ for K).

#### 3.4.4. Performance of the CARS-PLS Models

**Figure 4:**
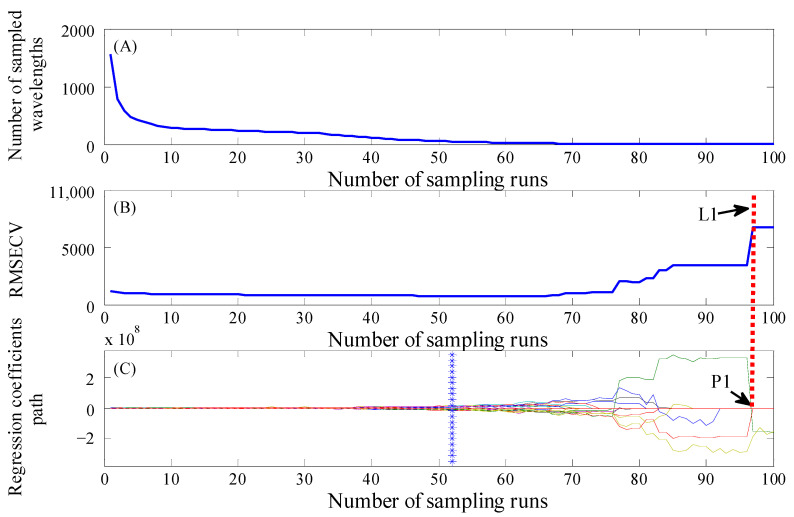
Plots of CARS wavelength selection on spectra data for Na. Plot (**A**–**C**) show the changing trend of the number of sampled wavelengths, *RMSECV* values, and the regression coefficient path of each wavelength with the increase in sampling runs, respectively. The line marked by blue asterisks in plot (**C**) represents the optimal point corresponding to the lowest *RMSECV* value in plot (**B**). The red dotted line marked as L1 denotes the sampling point at which the *RMSECV* value jumps to a higher stage. P1 denotes the coefficient of one key wavelength that drops to zero at the same sampling point. CARS = competitive adaptive reweighted sampling; *RMSECV* = root mean square error of cross-validation.

In CARS, Monte Carlo sampling was performed 100 times. The wavelength selection process of CARS is illustrated in Figure 4, using Na as an example. This demonstrated the changing trend in the number of selected wavelengths (Figure 4A), the *RMSECV* (Figure 4B) and regression coefficient paths (Figure 4C), along with the number of sampling runs. As depicted in Figure 4A,B, the selected wavelengths exhibited a rapid initial decrease followed by a gradual decline with an increase in sampling times. Meanwhile, the *RMSECV* initially decreased and subsequently showed an upward trend. This finding indicated that the wavelengths irrelevant to target information were continuously removed by CARS [39]. At the initial stage of sampling, a large number of irrelevant wavelengths were identified and deleted, which led to a rapid decrease in the *RMSECV* [25]. The *RMSECV* reached its minimum value at the 52nd sampling, indicating that the wavelength subset was considered optimal. However, after this point, the *RMSECV* gradually increased and rapidly multiplied by about 77 times. This suggested that important wavelengths related to target information were removed, resulting in a degradation of model performance. As shown in Figure 4C, the wavelengths with higher absolute values of regression coefficients were identified as key ones that should be retained, while those with lower absolute values were eliminated. The wavelength represented by P1, which had a regression coefficient approximating zero, was excluded. This exclusion resulted in an increase in the *RMSECV* and an immediate deterioration of the model’s performance, as indicated by L1 in Figure 4B. Finally, 50, 18, 36, 28, 20, and 54 key wavelengths were selected to develop the CARS-PLS model for Na, Mg, Ca, Cu, Fe, and K, respectively. Specifically, the wavelengths predominantly selected by the Na model were mainly distributed in the ranges of 1003–1177 nm, 1872–2053 nm, and 2195–2488 nm. As can be seen from Table 3, compared with other models, CARS-PLS models achieved the best predictions with higher *R_P_*^2^ and lower *RMSEP* values.

### 3.5. Discussion of Results

#### 3.5.1. Comparison of Different PLS Models

**Figure 5:**
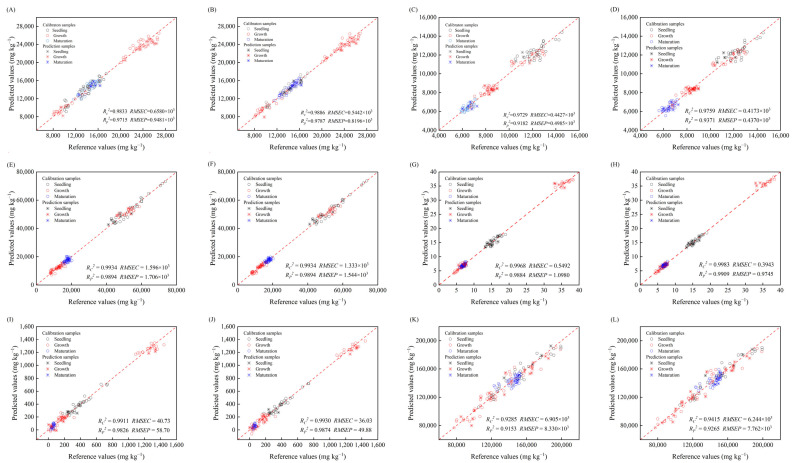
Reference values versus predicted values for Na (**A**,**B**), Mg (**C**,**D**), Ca (**E**,**F**), Cu (**G**,**H**), Fe (**I**,**J**), and K (**K**,**L**) and using full-range spectrum-partial least squares (Full-PLS) models (**A**,**C**,**E**,**G**,**I**,**K**) and competitive adaptive reweighted sampling-partial least squares (CARS-PLS) models (**B**,**D**,**F**,**H**,**J**,**L**). The samples in calibration and prediction sets were marked by the circles and asterisks, respectively. The samples for seedling, growth, and maturation stages were marked with blank, red, and blue, respectively. *R_C_*^2^ = coefficient of determination of calibration; *R_P_*^2^ = coefficient of determination of prediction; *RMSEC* = root mean square error of calibration; *RMSEP* = root mean square error of prediction.

The model performance of Na followed the order CARS-PLS > GA-PLS > SI-PLS > Full-PLS. In terms of Mg, Ca, Cu, Fe, and K, the preference order for PLS models was as follows: CARS-PLS > SI-PLS > GA-PLS > Full-PLS. This finding revealed the efficiency of the wavelength selection methods in the model optimization. Additionally, CARS outperformed the other PLS models, which is also demonstrated in Figure 5. Taking Na as an example, the wavelengths within the ranges of 1900–2053 nm and 2240–2302 nm were all selected by SI, GA, and CARS, illustrating that these wavelengths were key wavelengths for the quantitative analysis of Na. Compared to SI, GA and CARS included additional wavelengths at 1003–1177 nm and 2374–2488 nm, suggesting that these regions also play a pivotal role in the modeling. As an inorganic component, minerals do not contain functional groups such as C-H, O-H, resulting in no direct absorption peak in the NIR spectroscopy. However, numerous studies have demonstrated that in a mineral-rich environment, a series of corresponding changes occur in the growth and metabolism of algae. These changes specifically manifest as variations in proteins, lipids, and other substances, leading to– alterations in NIR spectroscopy, which can indirectly provide mineral information [40,41,42]. CARS can accurately and efficiently select informative wavelengths by employing ARS and EDF. However, SI-PLS cannot avoid the collinearity problem of the spectrum, and there are still some irrelevant wavelengths in the subset; the GA is prone to becoming trapped in local optimal solutions.

To demonstrate the merits of the CARS-PLS model with greater clarity, the scatter plots for the CARS-PLS and Full-PLS were constructed. The scatter plots that were closer to the dashed line (1:1) indicated better performance of the model. Compared with the Full-PLS model, the scatter plots of CARS-PLS exhibited a more concentrated distribution and a closer approximation to the dashed line, suggesting a stronger correlation. Specifically, the CARS-PLS models performed better in the prediction set, and the *R_P_*^2^ values increased from 0.9715, 0.9182, 0.9894, 0.9884, and 0.9826 in the Full-PLS models to 0.9787, 0.9371, 0.9913, 0.9909, and 0.9874 in the CARS-PLS models for Na, Mg, Ca, Cu, and Fe, respectively. The *RMSEP* values decreased remarkably from 0.9481 × 10^3^ mg kg^−1^, 0.4985 × 10^3^ mg kg^−1^, 1.706 × 10^3^ mg kg^−1^, 1.0980 mg kg^−1^, and 58.70 mg kg^−1^ in the Full-PLS models to 0.8196 × 10^3^ mg kg^−1^, 0.4370 × 10^3^ mg kg^−1^, 1.544 × 10^3^ mg kg^−1^, 0.9745 mg kg^−1^, and 49.88 mg kg^−1^ in the CARS-PLS models, respectively. As for K, this also achieved satisfactory improvement, even though the effect was not as remarkable as that of the above five indicators; the *R_P_*^2^ and *RMSEP* values also reached 0.9265 and 7.762 × 10^3^ mg kg^−1^, respectively.

#### 3.5.2. Comparison of Minerals of *S. fusiforme* at Different Growth Stages

**Figure 6:**
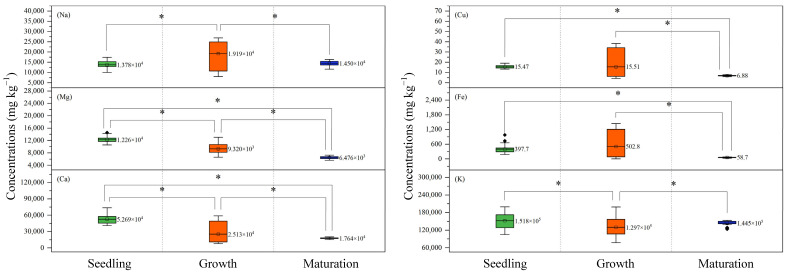
The minerals performance in different growth stages of *Sargassum fusiforme* for Na, Mg, Ca, Cu, Fe, and K. The number in the figure is the average value of mineral content of each growth stage. Seedling contains the first and second batch, Growth contains the third, fourth, and fifth batches, and Maturation contains the sixth and seventh batches. The asterisks indicate significant differences between variables (*p* < 0.05).

To further demonstrate the differences in the mineral concentration in *S. fusiforme* at different growth stages, the ANOVA was performed and the results were plotted in Figure 6. Asterisks indicate that the mineral was significantly different at a confidence level of *p* < 0.05. The mineral concentration of *S. fusiforme* exhibited significant variation across different growth stages. Specifically, the content of Mg and Ca exhibited a gradual decreasing trend, with the highest content observed during the seedling stage and the lowest in the maturation stage. At the *p* < 0.05 confidence level, significant differences were observed among different growth stages. The contents of Cu and Fe were the lowest in the maturation stage, being significantly lower than those in the seedling and growth stages. In the analysis of Na, the levels observed during the seedling and maturation stages were similar to each other and significantly lower than that observed in the growth stage. As for K, the analysis revealed no significant variation between the seedling stage and the maturation stage; however, both stages exhibited markedly higher levels compared to the growth stage. In conclusion, the seedling stage demonstrated the highest levels of Mg and Ca, and the maturation stage demonstrated the lowest levels of Cu and Fe. As for the growth stage, the concentration of Na was the highest, while that of K was the lowest.

## 4. Conclusions

The potential of NIR in the monitoring of Na, Mg, Ca, Cu, Fe, and K in *S. fusiforme* was demonstrated in this study. Four different spectral pretreatment methods including smooth, SNV, MSC, and 1D/SG were applied, and the PLS quantitative models were constructed. Three wavelength selection methods, namely SI, GA, and CARS, were performed for model optimization. CARS-PLS models exhibited superior performances with *R_P_*^2^ and *RMSEP* values of 0.9787 and 0.8196 × 10^3^ mg kg^−1^ for Na, 0.9371 and 0.4370 × 10^3^ mg kg^−1^ for Mg, 0.9913 and 1.544 × 10^3^ mg kg^−1^ for Ca, 0.9909 and 0.9745 mg kg^−1^ for Cu, 0.9874 and 49.88 mg kg^−1^ for Fe, and 0.9265 and 7.762 × 10^3^ mg kg^−1^ for K, respectively. The variations in the six mineral concentrations along the development stages were also investigated, and it was found that the seedling stage demonstrated the highest levels of Mg and Ca and the maturation stage demonstrated the lowest levels of Cu and Fe. Additionally, *S. fusiforme* showed the highest Na and the lowest K in the growth stage. In conclusion, NIR combined with CARS-PLS can rapidly and accurately predict the mineral levels in *S. fusiforme* at different developmental stages, which is helpful for the enhancement of the quality control level and guidance of the harvest strategies of *S. fusiforme*.

## Figures and Tables

**Table 1:** Statistical results of samples in the total, calibration, and prediction sets.

Sets	Concentration (mg kg^−1^)					
	Na (136)	Mg (135)	Ca (134)	Cu (133)	Fe (114)	K (135)
Total sets	16,300 ± 5291	9239 ± 2530	29,890 ± 19,230	12.90 ± 9.81	388.5 ± 433.8	139,800 ± 26,550
Calibration sets	15,820 ± 5096	9297 ± 2688	31,140 ± 19,610	12.41 ± 9.65	392.7 ± 431	138,500 ± 25,830
Prediction sets	18,220 ± 5618	9007 ± 1743	24,680 ± 16,570	14.94 ± 10.21	370.6 ± 444.9	145,200 ± 28,630

**Table 2:** Effects of different spectral pretreatment methods on the Full-PLS models.

Parameter	Model	LVs	Calibration			Prediction	
			*R_C_* ^2^	*RMSEC* (mg kg^−1^)	*RMSECV* (mg kg^−1^)	*R_P_* ^2^	*RMSEP* (mg kg^−1^)
Na	Raw	17	0.9771	0.7684 × 10^3^	1.074 × 10^3^	0.9673	1.027 × 10^3^
	Smooth	18	0.9774	0.7632 × 10^3^	1.066 × 10^3^	0.9659	1.048 × 10^3^
	SNV	14	0.9776	0.7910 × 10^3^	1.015 × 10^3^	0.9603	1.056 × 10^3^
	MSC	14	0.9779	0.7853 × 10^3^	1.013 × 10^3^	0.9602	1.058 × 10^3^
	1D/SG	11	0.9833	0.6580 × 10^3^	1.164 × 10^3^	0.9715	0.948 × 10^3^
Mg	Raw	13	0.9729	0.4427 × 10^3^	0.5294 × 10^3^	0.9182	0.4985 × 10^3^
	Smooth	13	0.9727	0.4439 × 10^3^	0.5264 × 10^3^	0.9179	0.4993 × 10^3^
	SNV	13	0.9776	0.3867 × 10^3^	0.4808 × 10^3^	0.9291	0.5849 × 10^3^
	MSC	14	0.9799	0.3667 × 10^3^	0.4871 × 10^3^	0.9266	0.5952 × 10^3^
	1D/SG	13	0.9954	0.1768 × 10^3^	0.5061 × 10^3^	0.9364	0.5540 × 10^3^
Ca	Raw	17	0.9934	1.596 × 10^3^	2.138 × 10^3^	0.9894	1.706 × 10^3^
	Smooth	17	0.9933	1.608 × 10^3^	2.078 × 10^3^	0.9888	1.751 × 10^3^
	SNV	18	0.9965	1.151 × 10^3^	1.903 × 10^3^	0.9895	1.813 × 10^3^
	MSC	18	0.9964	1.169 × 10^3^	1.956 × 10^3^	0.9870	2.016 × 10^3^
	1D/SG	15	0.9990	0.606 × 10^3^	2.098 × 10^3^	0.9833	2.149 × 10^3^
Cu	Raw	16	0.9940	0.7520	0.9975	0.9949	0.7323
	Smooth	16	0.9929	0.8182	1.0176	0.9939	0.7993
	SNV	13	0.9951	0.6760	0.8854	0.9843	1.2784
	MSC	16	0.9968	0.5492	0.8508	0.9884	1.0980
	1D/SG	10	0.9947	0.7359	1.1083	0.9919	0.7220
Fe	Raw	17	0.9908	41.37	68.56	0.9753	69.90
	Smooth	18	0.9911	40.73	66.96	0.9826	58.70
	SNV	18	0.9939	32.47	68.31	0.9843	60.50
	MSC	18	0.9945	30.98	68.57	0.9845	60.15
	1D/SG	5	0.9796	87.81	63.19	0.9770	58.77
K	Raw	18	0.9297	6.847 × 10^3^	1.046 × 10^4^	0.9128	8.457 × 10^3^
	Smooth	18	0.9285	6.905 × 10^3^	1.030 × 10^4^	0.9153	8.330 × 10^3^
	SNV	18	0.9462	5.836 × 10^3^	1.054 × 10^4^	0.9245	8.643 × 10^3^
	MSC	17	0.9354	6.393 × 10^3^	1.030 × 10^4^	0.9121	9.325 × 10^3^
	1D/SG	18	0.9977	1.284 × 10^3^	1.137 × 10^4^	0.8691	9.482 × 10^3^

SNV = standard normalized variate; MSC = multiplicative scatter correction; 1D/SG = the first derivative Savitzky–Golay; *R_C_*^2^ = coefficient of determination for calibration; *RMSEC* = root mean square error of calibration; *R_P_*^2^ = coefficient of determination for prediction; *RMSEP* = root mean square error of prediction; LVs = latent variables.

**Table 3:** Results of the PLS models based on different wavelength selection methods.

Parameter	Model	LVs	Variables	Calibration		Prediction	
				*R_C_* ^2^	*RMSEC* (mg kg^−1^)	*R_P_* ^2^	*RMSEP* (mg kg^−1^)
Na	Full-PLS	11	1557	0.9833	0.6580 × 10^3^	0.9715	0.9481 × 10^3^
	SI-PLS	13	328	0.9802	0.7171 × 10^3^	0.9764	0.8632 × 10^3^
	GA-PLS	12	92	0.9771	0.7716 × 10^3^	0.9768	0.8557 × 10^3^
	CARS-PLS	11	50	0.9886	0.5442 × 10^3^	0.9787	0.8196 × 10^3^
Mg	Full-PLS	13	1557	0.9729	0.4427 × 10^3^	0.9182	0.4985 × 10^3^
	SI-PLS	11	367	0.9728	0.4431 × 10^3^	0.9348	0.4451 × 10^3^
	GA-PLS	14	85	0.9766	0.4114 × 10^3^	0.9216	0.4881 × 10^3^
	CARS-PLS	11	18	0.9759	0.4173 × 10^3^	0.9371	0.4370 × 10^3^
Ca	Full-PLS	17	1557	0.9934	1.596 × 10^3^	0.9894	1.706 × 10^3^
	SI-PLS	17	347	0.9972	1.034 × 10^3^	0.9902	1.636 × 10^3^
	GA-PLS	17	103	0.9934	1.597 × 10^3^	0.9900	1.660 × 10^3^
	CARS-PLS	16	36	0.9954	1.333 × 10^3^	0.9913	1.544 × 10^3^
Cu	Full-PLS	16	1557	0.9968	0.5492	0.9884	1.0980
	SI-PLS	16	390	0.9974	0.4923	0.9903	1.0034
	GA-PLS	18	98	0.9973	0.4983	0.9897	1.0360
	CARS-PLS	17	28	0.9983	0.3943	0.9909	0.9745
Fe	Full-PLS	18	1557	0.9911	40.73	0.9826	58.70
	SI-PLS	17	415	0.9902	42.61	0.9873	50.08
	GA-PLS	18	85	0.9899	43.33	0.9849	54.59
	CARS-PLS	17	20	0.9930	36.03	0.9874	49.88
K	Full-PLS	18	1557	0.9285	6.905 × 10^3^	0.9153	8.330 × 10^3^
	SI-PLS	16	328	0.9217	7.229 × 10^3^	0.9190	8.149 × 10^3^
	GA-PLS	18	72	0.9099	7.754 × 10^3^	0.9169	8.251 × 10^3^
	CARS-PLS	15	54	0.9415	6.244 × 10^3^	0.9265	7.762 × 10^3^

PLS = partial least squares; Full-PLS = the PLS models constructed based on the full spectrum; SI-PLS = the PLS models constructed based on the wavelengths selected by synergy interval partial least square (SI-PLS) method; GA-PLS = the PLS models constructed based on the wavelengths selected by genetic algorithm (GA); CARS-PLS = the PLS models constructed based on the wavelengths selected by competitive adaptive reweighted sampling (CARS) method; *R_C_^2^* = coefficient of determination for calibration; *RMSEC* = root mean square error of calibration; *R_P_^2^* = coefficient of determination for prediction; *RMSEP* = root mean square error of prediction; LVs = latent variables.

**Table 4:** Results of SI-PLS models with selected optimal spectral subintervals for Na.

	Number of Subintervals	Selected Subintervals	LVs	*R_C_* ^2^	*RMSEC* (mg kg^−1^)	*R_P_* ^2^	*RMSEP* (mg kg^−1^)
Na	11	[2 3 7 9]	13	0.9860	0.6034 × 10^3^	0.9705	0.9654 × 10^3^
	12	[2 3]	13	0.9801	0.7198 × 10^3^	0.9744	0.8987 × 10^3^
	13	[2 3]	13	0.9795	0.7289 × 10^3^	0.9757	0.8750 × 10^3^
	14	[2 3 4]	12	0.9792	0.7354 × 10^3^	0.9763	0.8652 × 10^3^
	15	[2 3 4]	13	0.9795	0.7292 × 10^3^	0.9756	0.8783 × 10^3^
	16	[2 3 4 10]	12	0.9783	0.7515 × 10^3^	0.9760	0.8698 × 10^3^
	17	[3 4 5 11]	12	0.9816	0.6909 × 10^3^	0.9736	0.9134 × 10^3^
	18	[3 4 6 12]	12	0.9764	0.7826 × 10^3^	0.9761	0.8680 × 10^3^
	19	[2 3 4 6]	13	0.9802	0.7171 × 10^3^	0.9764	0.8632 × 10^3^
	20	[3 4 5 16]	13	0.9824	0.6764 × 10^3^	0.9730	0.9234 × 10^3^

SI-PLS = the PLS models constructed based on the wavelengths selected by synergy interval (SI) method; *R_C_*^2^ = coefficient of determination for calibration; *RMSEC* = root mean square error of calibration; *R_P_*^2^ = coefficient of determination for prediction; *RMSEP* = root mean square error of prediction; LVs = latent variables.

## Data Availability

The original contributions presented in the study are included in the article, further inquiries can be directed to the corresponding author.
